# Integral Representation of Electrostatic Interactions inside a Lipid Membrane

**DOI:** 10.3390/molecules25173824

**Published:** 2020-08-22

**Authors:** Guilherme Volpe Bossa, Sylvio May

**Affiliations:** 1 Department of Physics, São Paulo State University (UNESP), Institute of Biosciences, Humanities and Exact Sciences, São José do Rio Preto, SP 15054-000, Brazil; guilherme.vbossa@gmail.com; 2 Department of Physics, North Dakota State University, Fargo North Dakota, ND 58108-6050, USA

**Keywords:** Debye-Hückel, Bessel function, screened Coulomb potential, dielectric slab

## Abstract

Interactions between charges and dipoles inside a lipid membrane are partially screened. The screening arises both from the polarization of water and from the structure of the electric double layer formed by the salt ions outside the membrane. Assuming that the membrane can be represented as a dielectric slab of low dielectric constant sandwiched by an aqueous solution containing mobile ions, a theoretical model is developed to quantify the strength of electrostatic interactions inside a lipid membrane that is valid in the linear limit of Poisson-Boltzmann theory. We determine the electrostatic potential produced by a single point charge that resides inside the slab and from that calculate charge-charge and dipole-dipole interactions as a function of separation. Our approach yields integral representations for these interactions that can easily be evaluated numerically for any choice of parameters and be further simplified in limiting cases.

## 1. Introduction

Electrostatic interactions play a pivotal role inside and in the vicinity of every living cell [1,2,3]. They make essential contributions to the structure and functioning of all major types of biomacromolecules or biomacromolecular assemblies such as proteins, DNA, and lipid membranes. The corresponding description and understanding of electrostatic forces on the nanometer scale faces two major challenges. The first is the presence of salt ions in the aqueous medium, which leads to the formation of an electric double layer in the vicinity of a charged biomacromolecule. The second is the mismatch of the dielectric constant between the surrounding aqueous medium and the interior of a biomacromolecule [4]. A lipid membrane illustrates both features: It can be viewed as an extended dielectric slab that faces a salt-containing aqueous solution on each side. This introduces two length scales, the thickness *d* of the dielectric slab and the Debye screening length $l_{D}$, which characterizes the bulk salt concentration.

Many studies have been carried out that address electrostatic properties inside lipid membranes. Most of these studies are computational, based on Molecular Dynamics simulations [5,6,7], on implicit solvation models [8,9], or combinations of the two [10,11]. They are able to address specific questions like the energy cost of passing charges through the bilayer [12,13] or the interaction between transmembrane helices [14]. There are also non-computational models based on mean-field electrostatics [15] that are simple and thus allow to address fundamental questions such as the nature of electrostatic interactions of asymmetric membranes [16] and in ion channels [17,18,19,20,21], the electrostatic contribution to the bending stiffness of a lipid membrane [22,23], interactions of macroions across a lipid bilayer [24,25,26], or the stability of charged membrane domains [27,28]. The calculation of electrostatic interactions inside lipid bilayers using non-computational methods often leads to analytic expressions and thus to deeper insights into the underlying physical mechanisms. Yet, these calculations tend to be non-trivial and therefore often involve significant approximations, including the use of linear electrostatics, the representation of biomolecules by objects of simple symmetry [26], and the allocation of all polarization effects to the dielectric interfaces of the slab [29,30].

**Figure 1:**
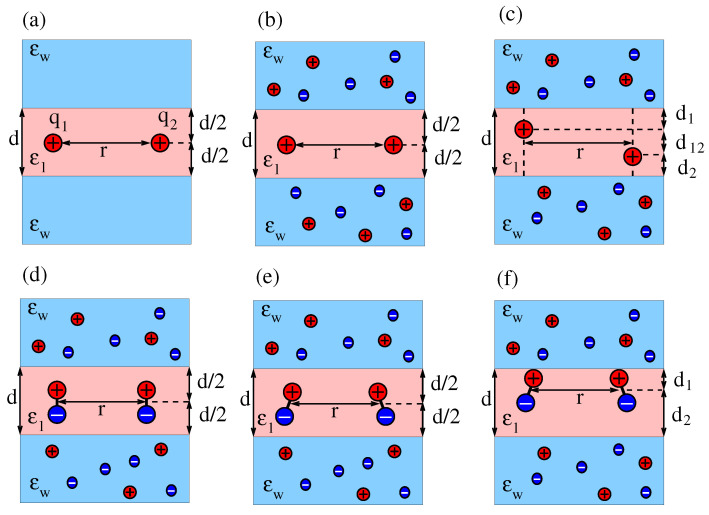
(**a**) Two interacting point charges, $q_{1}$ and $q_{2}$, separated by a distance *r* in the middle of a dielectric slab of dielectric constant $\epsilon_{l}$ and thickness *d*. The dielectric constant of the sandwiching media is $\epsilon_{w}$. (**b**) Salt ions with bulk concentration $n_{0}$ are present in the two sandwiching media. (**c**) The two interacting charges are moved up or down so that their mutual distance is $\sqrt{d_{12}^{2}+r^{2}}$ and their distances to the dielectric interfaces are $d_{1}$ and $d_{2}$, with $d_{1}+d_{2}+d_{12}=d$. (**d**) The two charges in diagram b are replaced by two dipoles, both located at the middle of and oriented normal to the dielectric slab, either parallel (as shown) or anti-parallel (not shown). (**e**) Two dipoles as in diagram d, yet with arbitrary orientations. (**f**) The two interacting dipoles shown in diagram e are jointly moved up or down so that their distances to the dielectric interfaces are $d_{1}$ and $d_{2}$, with $d_{1}+d_{2}=d$.

The present work is motivated by a study of Stillinger [31], who calculated the electrostatic potential of a point charge at the interface between a dielectric medium of low dielectric constant $\epsilon_{l}$ and a salt-containing aqueous solution of dielectric constant $\epsilon_{w}$. Application of Stillinger’s result to the interaction of two identical interfacial point charges by Hurd [32] revealed that the aqueous half-space mediates a screened contribution and the salt-free dielectric medium a dipolar contribution to the total interaction. Subsequent work has extended Stillinger’s approach to one or two point charges that are located above or below a dielectric interface, separating a medium with salt from another one without salt [33]. Here, we replace the dielectric half-space by a dielectric slab of thickness *d*. Indeed, a dielectric slab that is sandwiched by a salt-containing aqueous solution is a suitable electrostatic representation of a lipid bilayer. Our goal is to calculate the interaction between two point charges $q_{1}$ and $q_{2}$ and also between two dipoles with dipole moments $\mu_{1}$ and $\mu_{2}$ that are located inside the dielectric slab, thereby consistently accounting for the screening provided by the dielectric discontinuity and by the salt ions in the two sandwiching media. To make the development of our model transparent, we proceed in six steps that are illustrated in Figure 1.

We first consider the interaction between two point charges $q_{1}$ and $q_{2}$ that are separated by a distance *r* and are located in the middle of a dielectric slab of thickness *d* (see Figure 1a). Next, we add salt to the two sandwiching media (see Figure 1b) and, subsequently, allow for arbitrary locations of the two charges inside the dielectric slab (see Figure 1c). For the latter case, we characterize the locations of $q_{1}$ and $q_{2}$ such that their horizontal separation is *r* and, in addition, $q_{1}$ is a distance $d_{1}$ away from one interface and $q_{2}$ is a distance $d_{2}$ away from the other interface. The vertical distance between the two charges is then $d_{12}=d-d_{1}-d_{2}$, and their mutual distance is $\sqrt{d_{12}^{2}+r^{2}}$. We recognize that, while excess charges residing at or close to the interfacial region of a lipid bilayer are very common (charged lipid headgroups are examples), inserting them into the hydrocarbon chain region is associated with a high free energy cost [34,35] and is therefore not commonly encountered [36]. This is different for dipoles. Transmembrane helices possess non-vanishing dipoles that interact with each other [37]. We therefore exemplify the formalism developed in this work by calculating the interaction between two dipoles inside a dielectric slab. We first consider two dipoles that are both oriented normal to the dielectric slab and are located at the midplane (see Figure 1d). Then, we allow for arbitrary orientations of the two dipoles (see Figure 1e). Finally, we investigate a specific asymmetric case, where the two (arbitrarily oriented) dipoles both have the same distances $d_{1}$ and $d_{2}$ to the two interfaces (see Figure 1f). Note that we do not consider the most general case of dipoles with arbitrary location and orientation because this leads to cumbersome expressions. However, this and other scenarios (such as higher-order multipoles) can in principle be analyzed using the formalism developed in this work.

Our goal to derive explicit expressions for electrostatic interactions inside a lipid membrane relies on using linearized electrostatics, which requires the magnitude of the electrostatic potential everywhere in the salt-containing aqueous solution to be small. At physiological conditions this commonly requires the magnitude of the electrostatic potential to not exceed 25 mV [2]. This is problematic when considering point charges that are immersed directly into the aqueous solution. However, in our work we focus on point charges that are located inside the dielectric slab, which renders the use of linear electrostatics more appropriate. The same reasoning also applies to other approximations such as the neglect of dielectric saturation and ion–ion correlations outside the slab.

To summarize the goal of this work, we present a formalism to calculate interactions between charges and dipoles inside a lipid bilayer, valid on the level of linearized electrostatics and leading to integral representations that we discuss and analyze. Because of its linear nature, our method can in principle be applied to any interacting charge or multipole distributions inside a lipid membrane.

## 2. Theory and Analysis

### 2.1. Interaction between Two Point Charges

The interaction energy between two point charges $q_{1}$ and $q_{2}$ that are separated by a distance *r* and reside in a medium of uniform dielectric constant $\epsilon_{l}$ is $U=q_{1}q_{2}/\left(4\pi \epsilon_{0}\epsilon_{l}r\right)$, where $\epsilon_{0}$ is the permittivity of free space. Assume the two charges both reside in the middle between two large metal plates that form a parallel-plate capacitor with a plate-to-plate distance *d*. The two plates enforce a constant potential, which strongly screens the interaction between the two charges. For r≫d, the interaction can be approximated by the exponential screening $U=\alpha q_{1}q_{2}e^{-\pi r/d}/\left(4\pi \epsilon_{0}\epsilon_{l}d\right)$, where d/π serves as the screening length and α is a numerical factor of order one [38]. In the general case (valid for any choices of *d* and *r*), the interaction can be described by an infinite set of discrete image charges of alternating sign and separation *d*. The image charges are associated with one of the two point charges so that the other point charge interacts not only with its original partner but also with all of its images. This gives rise to the interaction energy [38]

(1) $$U=\frac{q_{1}q_{2}}{4\pi \epsilon_{0}\epsilon_{l}}\sum_{n=-\infty }^{\infty }\frac{{\left(-1\right)}^{n}}{\sqrt{r^{2}+{\left(nd\right)}^{2}}}=\frac{q_{1}q_{2}}{4\pi \epsilon_{0}\epsilon_{l}}\left[\frac{1}{r}+2\sum_{n=1}^{\infty }\frac{{\left(-1\right)}^{n}}{\sqrt{r^{2}+{\left(nd\right)}^{2}}}\right].$$

An alternative description of this system places the two point charges (which are separated by a distance *r*) in the middle of a dielectric slab with dielectric constant $\epsilon_{l}$ that is sandwiched on each side by a medium of dielectric constant $\epsilon_{w}$; see Figure 1a. If $\epsilon_{w}$ is very large ($\epsilon_{w}\to \infty$), the two dielectric interfaces act like metal plates and thus produce the same interaction as in Equation (1). In the general case of $1\leq \epsilon_{w}\leq \infty$ we merely need to adjust the strength of the image charges from −1 to the factor $w=\left(\epsilon_{l}-\epsilon_{w}\right)/\left(\epsilon_{l}+\epsilon_{w}\right)$. The interaction energy between the two point charges then becomes [39,40,41]

(2) $$U=\frac{q_{1}q_{2}}{4\pi \epsilon_{0}\epsilon_{l}}\left[\frac{1}{r}+2\sum_{n=1}^{\infty }\frac{w^{n}}{\sqrt{r^{2}+{\left(nd\right)}^{2}}}\right].$$

An equivalent representation of Equation (2) can be obtained by making use of the identity $\int_{0}^{\infty }dkJ_{0}\left(kr\right)e^{-nkd}=1/\sqrt{r^{2}+{\left(nd\right)}^{2}}$, where $J_{0}$ denotes the Bessel function of the first kind and zeroth order, and the sum $1+2\sum_{n=1}^{\infty }w^{n}e^{-nkd}=\left(e^{kd}+w\right)/\left(e^{kd}-w\right)$. This gives rise to a simple integral representation for the interaction between the two point charges inside the dielectric slab

(3) $$U=\frac{q_{1}q_{2}}{4\pi \epsilon_{0}\epsilon_{l}}\int_{0}^{\infty }dk\;J_{0}\left(kr\right)\left[1+2\sum_{n=1}^{\infty }w^{n}e^{-nkd}\right]=\frac{q_{1}q_{2}}{4\pi \epsilon_{0}\epsilon_{l}}\int_{0}^{\infty }dk\;J_{0}\left(kr\right)\left[\frac{e^{kd}+w}{e^{kd}-w}\right].$$

We conclude our analysis of the case displayed in Figure 1a by noting that, as expected, Equation (3) reproduces the two limits $U=q_{1}q_{2}/\left(4\pi \epsilon_{0}\epsilon_{w}r\right)$ for d=0 and $U=q_{1}q_{2}/\left(4\pi \epsilon_{0}\epsilon_{l}r\right)$ for d→∞.

Next, as shown in Figure 1b, we identify the two outer regions of dielectric constant $\epsilon_{w}$ as a medium that hosts salt of bulk concentration $n_{0}$. This introduces another length scale, the Debye length $l_{D}={\left(8\pi l_{B}n_{0}\right)}^{-1/2}$, expressed in terms of the Bjerrum length $l_{B}=e^{2}/\left(4\pi \epsilon_{0}\epsilon_{w}k_{B}T\right)$, where *e* is the elementary charge, $k_{B}$ Boltzmann’s constant, and *T* the absolute temperature. The presence of the salt ions provides for additional screening of the electrostatic interaction between the two charges $q_{1}$ and $q_{2}$ that are inserted in the middle of the dielectric slab. We show in Appendix A and Appendix B that within the framework of linearized electrostatics, where the electrostatic potential Φ fulfills the equation $l_{D}^{2}\nabla^{2}\Phi =\Phi$ in the medium with dielectric constant $\epsilon_{w}$ and the equation $\nabla^{2}\Phi =0$ in the medium with dielectric constant $\epsilon_{l}$, the interaction energy between the two point charges $q_{1}$ and $q_{2}$ becomes

(4) $$U=\frac{q_{1}q_{2}}{4\pi \epsilon_{0}\epsilon_{l}}\int_{0}^{\infty }dk\;J_{0}\left(kr\right)\frac{{\left[\epsilon_{l}kl_{D}\cosh\left(\frac{kd}{2}\right)+\epsilon_{w}\sqrt{1+k^{2}l_{D}^{2}}\sinh\left(\frac{kd}{2}\right)\right]}^{2}}{\epsilon_{l}\epsilon_{w}kl_{D}\sqrt{1+k^{2}l_{D}^{2}}\cosh\left(kd\right)+\frac{1}{2}\left[\epsilon_{w}^{2}+\left(\epsilon_{l}^{2}+\epsilon_{w}^{2}\right)k^{2}l_{D}^{2}\right]\sinh\left(kd\right)}.$$

We reiterate that, as illustrated in Figure 1b, the two point charges are still located in the middle of the dielectric slab, each with distances d/2 to the two interfaces. The separation between the two charges is *r*. In the limit $n_{0}\to 0$, the Debye length becomes large, $l_{D}\to \infty$, and Equation (4) indeed recovers the result in Equation (3). In the opposite limit, $n_{0}\to \infty$, the Debye length vanishes, $l_{D}\to 0$, and the two dielectric interfaces become “metallic”. Equation (4) then recovers Equation (1). If the thickness of the dielectric slab grows very large, d→∞, we recover the Coulomb interaction $U=q_{1}q_{2}/\left(4\pi \epsilon_{0}\epsilon_{l}r\right)$. In contrast, in the limit d=0, the two charges are embedded in a salt-containing medium, which produces the familiar screened Coulomb interaction

(5) $$U=\frac{q_{1}q_{2}}{4\pi \epsilon_{0}\epsilon_{l}}\int_{0}^{\infty }dk\;J_{0}\left(kr\right)\frac{{\left(\epsilon_{l}kl_{D}\right)}^{2}}{\epsilon_{l}\epsilon_{w}kl_{D}\sqrt{1+k^{2}l_{D}^{2}}}=\frac{q_{1}q_{2}}{4\pi \epsilon_{0}\epsilon_{w}}\frac{e^{-r/l_{D}}}{r}.$$

**Figure 2:**
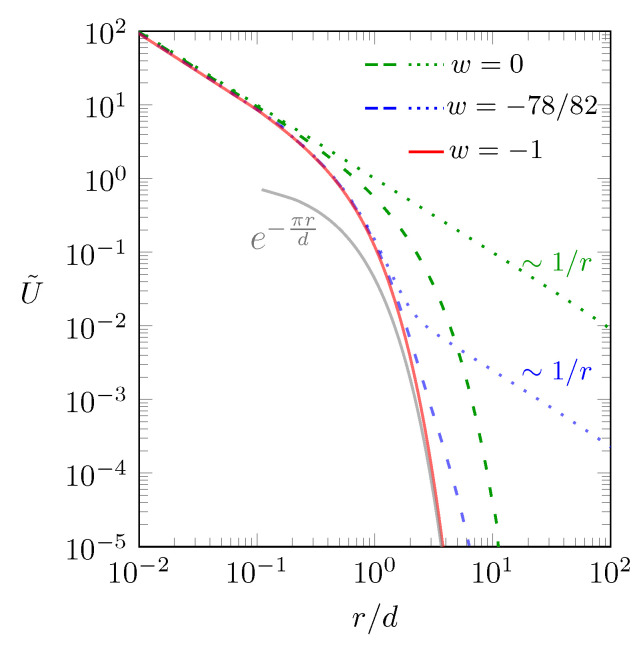
Scaled interaction energy $\tilde{U}=\left(4\pi \epsilon_{0}\epsilon_{l}d/q_{1}q_{2}\right)\times U$ between two point charges $q_{1}$ and $q_{2}$ as function of the scaled separation r/d according to Equation (4). Different curves corresponds to: w=−1 (red line); w=0 and $l_{D}\to \infty$ (green dotted line); w=0 and $l_{D}=d$ (green dashed line); w=−78/82 and $l_{D}\to \infty$ (blue dotted line); w=−78/82 and $l_{D}=d$ (blue dashed line). The gray line shows the approximation $\tilde{U}=e^{-\pi r/d}$ of the red line, valid in the limit r≫d [38]. Note that in all cases the two charges are located in the middle of the dielectric slab, $d_{1}=d_{2}=d/2$.

In Figure 2 we show the behavior of the scaled interaction energy $\tilde{U}=\left(4\pi \epsilon_{0}\epsilon_{l}d/q_{1}q_{2}\right)\times U$ as function of r/d according to Equation (4).

The red solid line describes the interaction in the “metallic” limit w=−1, when the screening is maximal. Recall the definition $w=\left(\epsilon_{l}-\epsilon_{w}\right)/\left(\epsilon_{l}+\epsilon_{w}\right)$; so this corresponds to $\epsilon_{w}\to \infty$: The two charges reside between two metal plates as introduced in Equation (1). We cannot carry out the summation in Equation (1) explicitly, but the expression $\tilde{U}\approx e^{-\pi r/d}$ approximates the limit r≫d [38], shown as the gray solid line in Figure 2. In the other limit, w=0, there is no dielectric mismatch and all screening is due to the salt. This is shown by the green lines in Figure 2. The green dotted line refers to the limit $l_{D}\to \infty$, where no salt is present and the interaction $U∼r^{-1}$ is Coulombic. The green dashed line refers to $l_{D}=d$. Here, the interaction is Coulombic for small and screened for large distances. When more salt is added, screening increases and the behavior approaches that of the red line (the “metallic” case as in Equation (1)). The red line describes the behavior for $l_{D}\to 0$, irrespective of *w*. We finally show one case in between the two limits w=−1 and w=0: The two blue lines refer to w=(2−80)/(2+80)=−78/82=−0.951, which is motivated by the typical dielectric constants $\epsilon_{w}\approx 80$ and $\epsilon_{l}\approx 2$. The blue dotted line describes the absence of salt, $l_{D}\to \infty$. Here, the interaction is Coulombic for small and large distances and screened in between. Adding salt moves the curve towards the red line; which is shown by the blue dashed line, referring to $l_{D}=d$. This concludes our analysis of the case displayed in Figure 1b.

So far, we have considered the two point charges $q_{1}$ and $q_{2}$ to be located exactly in the middle of the dielectric slab, with distances d/2 to each of the two interfaces. Next, we extend our model to the case that $q_{1}$ and $q_{2}$ are located anywhere inside the dielectric slab as shown in Figure 1c. We denote the lateral charge-to-charge distance by *r*, the vertical charge-to-charge distance by $d_{12}$, the distance of $q_{1}$ to its nearest interface by $d_{1}$, and the distance of $q_{2}$ to the other interface by $d_{2}$. This implies $d=d_{1}+d_{2}+d_{12}$ is the thickness of the dielectric slab, and $\sqrt{d_{12}^{2}+r^{2}}$ is the charge-to-charge distance. As we detail in Appendix A and Appendix B, we obtain for the interaction energy

(6) $$\begin{array}{ccc} U & = & \frac{q_{1}q_{2}}{4\pi \epsilon_{l}\epsilon_{0}}\int_{0}^{\infty }dk\;J_{0}\left(kr\right)\times \\ & \times & \frac{\left[\epsilon_{l}kl_{D}\cosh\left(kd_{1}\right)+\epsilon_{w}\sqrt{1+k^{2}l_{D}^{2}}\sinh\left(kd_{1}\right)\right]\times \left[\epsilon_{l}kl_{D}\cosh\left(kd_{2}\right)+\epsilon_{w}\sqrt{1+k^{2}l_{D}^{2}}\sinh\left(kd_{2}\right)\right]}{\epsilon_{l}\epsilon_{w}kl_{D}\sqrt{1+k^{2}l_{D}^{2}}\cosh\left(kd\right)+\frac{1}{2}\left[\epsilon_{w}^{2}+\left(\epsilon_{l}^{2}+\epsilon_{w}^{2}\right)k^{2}l_{D}^{2}\right]\sinh\left(kd\right)}. \end{array}$$

Equation (6) is a general expression, valid within linearized electrostatics, for the interaction energy of two point charges $q_{1}$ and $q_{2}$ inside a dielectric slab that is sandwiched by an electrolyte. It can be used to derive interaction energies between more complex charge distributions as we demonstrate below for two interacting dipoles. Equation (6) thus constitutes the primary result of the present work.

Of course, for $d_{1}=d_{2}=d/2$ and thus $d_{12}=0$, Equation (6) recovers Equation (4). In the limit $n_{0}\to 0$ the Debye length becomes large, and we expect to recover the interaction energy between two charges in a dielectric slab. We obtain from Equation (6) in the limit $l_{D}\to \infty$

(7) $$\begin{array}{ccc} U & = & \frac{q_{1}q_{2}}{4\pi \epsilon_{l}\epsilon_{0}}\int_{0}^{\infty }dk\;J_{0}\left(kr\right)\frac{\cosh\left[k\left(d_{1}+d_{2}\right)-\ln w\right]+\cosh\left[k\left(d_{1}-d_{2}\right)\right]}{\sinh[kd-\ln w]}\\ & = & \frac{q_{1}q_{2}}{4\pi \epsilon_{l}\epsilon_{0}}\{\;\frac{1}{\sqrt{r^{2}+d_{12}^{2}}}\;+2\;\;\;\;\sum_{n=2,4,\ldots }^{\infty }\;\;\frac{w^{n}}{\sqrt{r^{2}+{\left(nd+d_{12}\right)}^{2}}}\\ & + & \sum_{n=1,3,\ldots }^{\infty }\;\left[\;\frac{w^{n}}{\sqrt{r^{2}+{\left(nd+2d_{1}-d\right)}^{2}}}\;+\;\frac{w^{n}}{\sqrt{r^{2}+{\left(nd+2d_{2}-d\right)}^{2}}}\right]\}, \end{array}$$

which indeed reproduces the interaction energy between the two charges according to the image charge method. If the two interfaces become “metallic” (that is, in the limit $\epsilon_{w}\to \infty$ or $l_{D}\to 0$), Equation (6) reads

(8) $$U=\frac{q_{1}q_{2}}{2\pi \epsilon_{l}\epsilon_{0}}\int_{0}^{\infty }dk\;J_{0}\left(kr\right)\frac{\sinh\left(kd_{1}\right)\sinh\left(kd_{2}\right)}{\sinh(kd)}.$$

Of course, for $d_{1}=d_{2}=d/2$ we recover Equation (3) with w=−1, which is identical to Equation (1). In the limit d→0, Equation (6) recovers the screened interaction in Equation (5). Of interest is also the case $d_{1}=d_{12}=0$ and $d_{2}\to \infty$, where the two interacting charges are located at an interface between a medium of dielectric constant $\epsilon_{l}$ and another salt-containing medium of dielectric constant $\epsilon_{w}$. From Equation (6) we obtain

(9) $$U=\frac{q_{1}q_{2}}{4\pi \epsilon_{0}}\int_{0}^{\infty }dk\;J_{0}\left(kr\right)\frac{2kl_{D}}{\epsilon_{l}kl_{D}+\epsilon_{w}\sqrt{1+k^{2}l_{D}^{2}}},$$

which recovers the result first derived by Stillinger [31]. In the limit $\epsilon_{w}\gg \epsilon_{l}$, Hurd [32] decomposed Equation (9) into a screened and an unscreened dipolar contribution. The decomposition emerges upon expanding the integrand of Equation (9) up to first order in $\epsilon_{l}/\epsilon_{w}$,

(10) $$U=\frac{q_{1}q_{2}}{2\pi \epsilon_{0}\epsilon_{w}}\left(\frac{e^{-r/l_{D}}}{r}-\frac{\epsilon_{l}}{\epsilon_{w}}\frac{l_{D}^{2}}{r^{3}}\int_{0}^{\infty }dk\;J_{0}\left(k\right)\frac{k^{2}}{1+k^{2}\frac{l_{D}^{2}}{r^{2}}}\right)=\frac{q_{1}q_{2}}{2\pi \epsilon_{0}\epsilon_{w}}\left(\frac{e^{-r/l_{D}}}{r}+\frac{\epsilon_{l}}{\epsilon_{w}}\frac{l_{D}^{2}}{r^{3}}\right).$$

**Figure 3:**
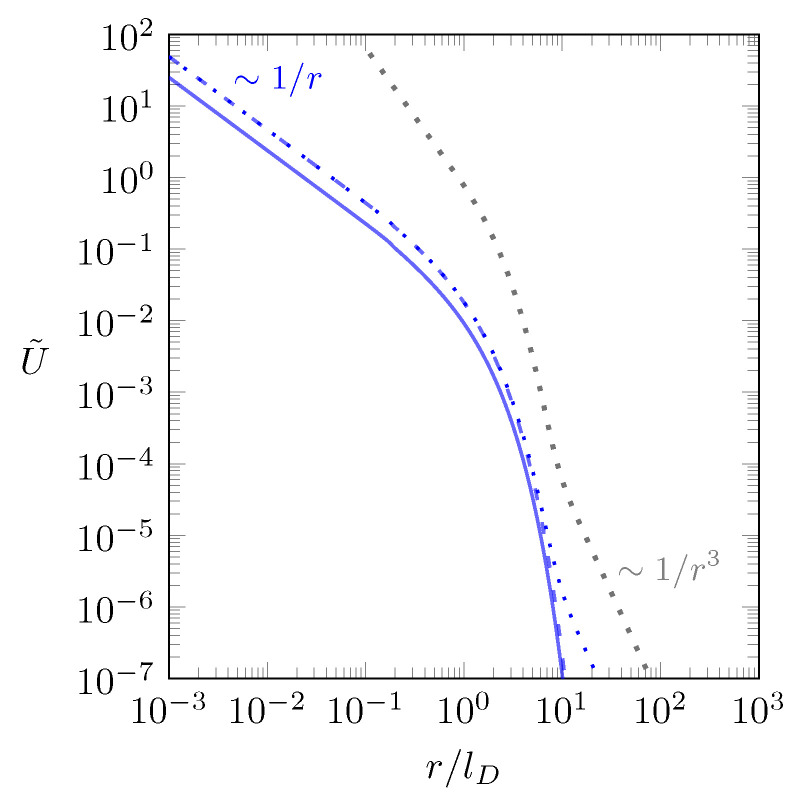
Scaled interaction energy $\tilde{U}=\left(4\pi \epsilon_{0}\epsilon_{l}l_{D}/q_{1}q_{2}\right)\times U$ between two point charges $q_{1}$ and $q_{2}$ that are both attached to the same interface ($d_{1}=0$ and $d_{2}=d$) as function of the scaled separation $r/l_{D}$ for w=−78/82. The blue lines correspond to d→∞ (dotted line), $d=l_{D}$ (dashed line), and d→0 (solid line), all calculated according to Equation (6). The gray dotted line is Hurd’s [32] decomposition specified in Equation (10).

Note that solving the integral producing the dipolar contribution in Equation (10) is based on the additional assumption $r\gg l_{D}$ [32]. In Figure 3 we illustrate how the dipolar contribution to the interaction emerges for two point charges that are both attached to the same interface ($d_{1}=0$ and $d_{2}=d$).

We consider the case w=−78/82, where there is a large dielectric mismatch between slab and surrounding medium and display the scaled interaction energy $\tilde{U}=\left(4\pi \epsilon_{0}\epsilon_{l}l_{D}/q_{1}q_{2}\right)\times U$ between two interface-attached point charges $q_{1}$ and $q_{2}$ as function of the scaled separation $r/l_{D}$, calculated according to Equation (6). The blue solid line applies to the limit d→0, where we simply obtain the screened Coulomb energy $\tilde{U}=\left(l_{D}/r\right)\times e^{-r/l_{D}}$; see Equation (5). The result obtained upon increasing the slab thickness to $d=l_{D}$ is shown as blue dashed line. Because the increased slab thickness diminishes the screening, the interaction energy increases, but it does increasingly less so for larger *r*. For sufficiently large *r* the interaction becomes identical to the result for d→0, and no dipolar contribution is present. Only in the limit d→∞ (blue dotted line) does a dipolar contribution emerge for sufficiently large *r*, and this contribution is indeed qualitatively reproduced by Hurd’s [32] decomposition specified in Equation (10) (shown as gray dotted line in Figure 3). The absence of a dipolar contribution for finite *d* is a consequence of the slab geometry ultimately allowing screening from both sides, irrespective of $d_{1}$ and $d_{2}$ (and also irrespective of $d_{12}$).

### 2.2. Interaction between Two Dipoles

As an application of the method developed in the previous section, we replace the two interacting point charges by dipoles with dipole moment $\mu_{1}$ and $\mu_{2}$, located at and oriented normal to the midplane (see Figure 1d). Note that a parallel orientation leads to a strictly repulsive interaction [42] (as opposed to the antiparallel orientation which is attractive). We model a dipole by two opposite charges, $q_{1,2}$ and $-q_{1,2}$, that are separated by a sufficiently small distance *l*, implying $\mu_{1}=q_{1}l$ and $\mu_{2}=q_{2}l$. If the two dipoles were located in a uniform medium of dielectric constant $\epsilon_{l}$, the interaction energy would be $U=\mu_{1}\mu_{2}/\left(4\pi \epsilon_{0}\epsilon_{l}r^{3}\right)$. The presence of the dielectric slab modifies that interaction in the following manner

(11) $$U=\frac{-\mu_{1}\mu_{2}}{4\pi \epsilon_{0}\epsilon_{l}}\lim_{\delta \to 0}\int_{0}^{\infty }dk\;J_{0}\left(kr\right)\;\frac{e^{-k\delta }\;k^{2}\;{\left[\epsilon_{l}kl_{D}\cosh\left(\frac{kd}{2}\right)+\epsilon_{w}\sqrt{1+k^{2}l_{D}^{2}}\sinh\left(\frac{kd}{2}\right)\right]}^{2}}{\epsilon_{l}\epsilon_{w}kl_{D}\sqrt{1+k^{2}l_{D}^{2}}\cosh\left(kd\right)+\frac{1}{2}\left[\epsilon_{w}^{2}+\left(\epsilon_{l}^{2}+\epsilon_{w}^{2}\right)k^{2}l_{D}^{2}\right]\sinh\left(kd\right)}.$$

Note the similarity of Equations (11) and (4), and also note the difference of the additional factor $k^{2}$ in the integrand. This additional factor necessitates the presence of the term $e^{-k\delta }$ in the integrand together with the limit δ→0 after carrying out the integration. Appendix C details the pathway to derive Equation (11). Let us verify the two limits of infinitely large and vanishing slab thickness. In the limit d→∞ we obtain

(12) $$U\left(r\right)=-\frac{\mu_{1}\mu_{2}}{4\pi \epsilon_{0}\epsilon_{l}}\lim_{\delta \to 0}\int_{0}^{\infty }dk\;e^{-k\delta }\;k^{2}J_{0}\left(kr\right)=-\frac{\mu_{1}\mu_{2}}{4\pi \epsilon_{0}\epsilon_{l}}\;\frac{1}{r^{3}}\;\lim_{\delta \to 0}\frac{2\delta^{2}-1}{{\left(1+\delta^{2}\right)}^{5/2}}=\frac{\mu_{1}\mu_{2}}{4\pi \epsilon_{0}\epsilon_{l}}\;\frac{1}{r^{3}},$$

and the limit d→0 yields

(13) $$U\left(r\right)=-\frac{\mu_{1}\mu_{2}}{4\pi \epsilon_{0}\epsilon_{w}}\lim_{\delta \to 0}\int_{0}^{\infty }dk\;e^{-k\delta }\;k\;\sqrt{k^{2}+l_{D}^{-2}}\;J_{0}\left(kr\right)=\frac{\mu_{1}\mu_{2}}{4\pi \epsilon_{0}\epsilon_{w}}\frac{1}{r^{3}}\left(1+\frac{r}{l_{D}}\right)e^{-r/l_{D}}.$$

**Figure 4:**
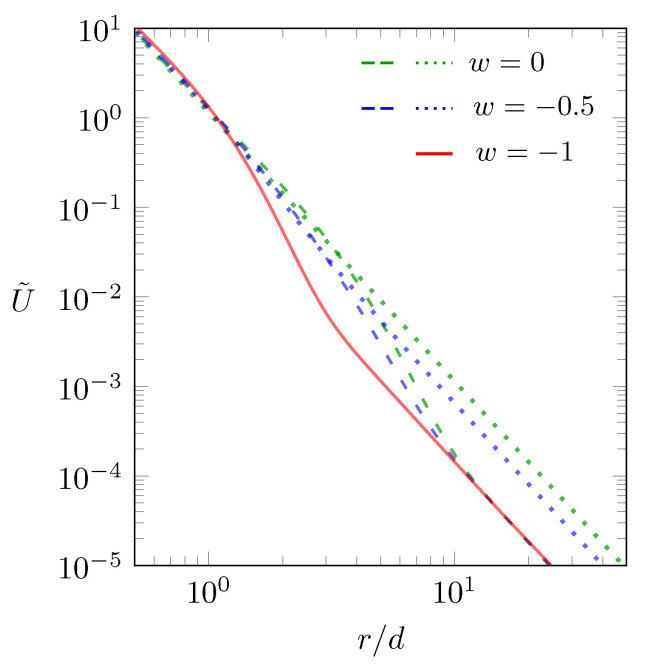
Scaled interaction energy $\tilde{U}=\left(4\pi \epsilon_{0}\epsilon_{l}d^{3}/\mu_{1}\mu_{2}\right)\times U$ between two dipoles as function of the scaled separation r/d, calculated according to Equation (11). The green dotted line refers to w=0 and $l_{D}\to \infty$, the green dashed line to w=0 and $l_{D}=d$, the blue dotted line to w=−0.5 and $l_{D}\to \infty$, and the blue dashed line to w=−0.5 and $l_{D}=d$. The “metallic” case ($l_{D}\to 0$) is shown by the red line, which is independent of *w*.

Both expressions agree with the familiar interaction between two dipoles that are aligned in one plane and are oriented perpendicular to the direction that connects them, one in the absence (Equation (12)) and the other in the presence (Equation (13)) of salt. The scaled interaction energy $\tilde{U}=\left(4\pi \epsilon_{0}\epsilon_{l}d^{3}/\mu_{1}\mu_{2}\right)\times U$ between two dipoles placed at the middle of the dielectric slab is displayed in Figure 4 according to Equation (11) as function of the scaled separation r/d.

The green dotted line shows $\tilde{U}$ for w=0 and $l_{D}\to \infty$. In this case, with no dielectric mismatch and no salt, the interaction is just $\tilde{U}={\left(d/r\right)}^{3}$. Adding salt, with $l_{D}=d$ while keeping w=0, introduces some screening and thus lowers the interaction when *r* is sufficiently large, as is shown by the green dashed line in Figure 4. Note that the $∼r^{-3}$ behavior persists for small *r* and for large *r*, the latter with a reduced magnitude due to the screening. The limit of adding an infinite amount of salt ($l_{D}\to 0$) is shown by the red line. This is the case of maximal screening (the “metallic” case), the same as if the two dipoles resided in between two metal plates. We have also calculated the same scenario (progressing from no salt to the hypothetical limit of an infinite amount of salt) for w=−0.5, which is shown by the blue dotted line (for $l_{D}\to \infty$) and by the blue dashed line (for $l_{D}\to d$). Here too, the scaling in all cases is $\tilde{U}∼r^{-3}$, both for small and large *r*. For non-vanishing dielectric mismatch or any non-vanishing salt content, $\tilde{U}$ will transition into the “metallic” case (the red line) for sufficiently large *r*.

Next, we allow the two dipoles to adopt arbitrary orientations while still being located at the midplane of the dielectric slab, as shown in Figure 1e. We characterize the orientation of the first dipole by its angle $\theta_{1}$ with respect to the *r*-direction (that is, the horizontal direction, parallel to the slab) and the azimuthal angle $\phi_{1}$. Angles $\theta_{2}$ and $\phi_{2}$ are introduced analogously for the second dipole. With this, the dipole–dipole interaction energy becomes

(14) $$\begin{array}{ccc} U & = & \frac{\mu_{1}\mu_{2}}{4\pi \epsilon_{0}\epsilon_{l}}\lim_{\delta \to 0}\int_{0}^{\infty }dk\;\left\{kJ_{0}\left(kr\right)\left[\cos\theta_{1}\cos\theta_{2}-\cos\left(\phi_{1}-\phi_{2}\right)\sin\theta_{1}\sin\theta_{2}\right]-\frac{J_{1}\left(kr\right)}{r}\cos\theta_{1}\cos\theta_{2}\right\}\;\times \\ & \times & \frac{e^{-k\delta }\;k\;{\left[\epsilon_{l}kl_{D}\cosh\left(\frac{kd}{2}\right)+\epsilon_{w}\sqrt{1+k^{2}l_{D}^{2}}\sinh\left(\frac{kd}{2}\right)\right]}^{2}}{\epsilon_{l}\epsilon_{w}kl_{D}\sqrt{1+k^{2}l_{D}^{2}}\cosh\left(kd\right)+\frac{1}{2}\left[\epsilon_{w}^{2}+\left(\epsilon_{l}^{2}+\epsilon_{w}^{2}\right)k^{2}l_{D}^{2}\right]\sinh\left(kd\right)}. \end{array}$$

In Appendix D we sketch the derivation of Equation (14). Obviously, Equation (11) is recovered from Equation (14) for $\theta_{1}=\theta_{2}=\pi /2$ and $\phi_{1}=\phi_{2}$. In the limit d→∞ we obtain from Equation (14)

(15) $$\begin{array}{c} U=\frac{\mu_{1}\mu_{2}}{4\pi \epsilon_{0}\epsilon_{l}}\;\frac{1}{r^{3}}\;\left[\cos\left(\phi_{1}-\phi_{2}\right)\sin\theta_{1}\sin\theta_{2}-2\cos\theta_{1}\cos\theta_{2}\right], \end{array}$$

which is identical to the familiar interaction energy $U=\left[{\vec{\mu }}_{1}\cdot {\vec{\mu }}_{2}-3\left({\vec{\mu }}_{1}\cdot {\vec{e}}_{12}\right)\cdot \left({\vec{\mu }}_{2}\cdot {\vec{e}}_{12}\right)\right]/\left(4\pi \epsilon_{0}\epsilon_{l}r^{3}\right)$ of two dipoles with moments $\mu_{1}$ and $\mu_{2}$, and orientations ${\vec{\mu }}_{1}=\mu_{1}\left\{\sin\theta_{1}\cos\phi_{1},\sin\theta_{1}\sin\phi_{1},\cos\theta_{1}\right\}$ as well as ${\vec{\mu }}_{2}=\mu_{2}\left\{\sin\theta_{2}\cos\phi_{2},\sin\theta_{2}\sin\phi_{2},\cos\theta_{2}\right\}$, where ${\vec{e}}_{12}=\left\{0,0,1\right\}$ is a unit vector pointing from one to the other dipole [43]. In the other limit, d→0, Equation (14), yields the screened dipole–dipole interaction

(16) $$U\left(r\right)=\frac{\mu_{1}\mu_{2}}{4\pi \epsilon_{0}\epsilon_{w}}\frac{1}{r^{3}}\left(1+\frac{r}{l_{D}}\right)e^{-r/l_{D}}\left[\cos\left(\phi_{1}-\phi_{2}\right)\sin\theta_{1}\sin\theta_{2}-2\cos\theta_{1}\cos\theta_{2}\right].$$

Our final result is for the interaction of two dipoles, with moments $\mu_{1}$ and $\mu_{2}$, of arbitrary orientation, again characterized by the angles $\theta_{1}$ and $\phi_{1}$ for the first and $\theta_{2}$ and $\phi_{2}$ for the second dipole. The dipoles reside inside the dielectric slab, both with distances $d_{1}$ and $d_{2}$ to the two interfaces (see Figure 1f). In this case the interaction energy is

(17) $$\begin{array}{ccc} U & = & \frac{\mu_{1}\mu_{2}}{4\pi \epsilon_{0}\epsilon_{l}}\lim_{\delta \to 0}\int_{0}^{\infty }dk\;\left\{kJ_{0}\left(kr\right)\left[\cos\theta_{1}\cos\theta_{2}-\cos\left(\phi_{1}-\phi_{2}\right)\sin\theta_{1}\sin\theta_{2}\right]-\frac{J_{1}\left(kr\right)}{r}\cos\theta_{1}\cos\theta_{2}\right\}\;\times \\ & \times & e^{-k\delta }\;k\;\\ & \times & \frac{\left[\epsilon_{l}kl_{D}\cosh\left(kd_{1}\right)+\epsilon_{w}\sqrt{1+k^{2}l_{D}^{2}}\sinh\left(kd_{1}\right)\right]\times \left[\epsilon_{l}kl_{D}\cosh\left(kd_{2}\right)+\epsilon_{w}\sqrt{1+k^{2}l_{D}^{2}}\sinh\left(kd_{2}\right)\right]}{\epsilon_{l}\epsilon_{w}kl_{D}\sqrt{1+k^{2}l_{D}^{2}}\cosh\left(kd\right)+\frac{1}{2}\left[\epsilon_{w}^{2}+\left(\epsilon_{l}^{2}+\epsilon_{w}^{2}\right)k^{2}l_{D}^{2}\right]\sinh\left(kd\right)}. \end{array}$$

Note that Equation (17) is still not the most general expression for two interacting dipoles inside a dielectric slab because we require both dipoles have the same distances to the two interfaces (see Figure 1f). Writing down the most general result is possible but the expression appears cumbersome and is thus not included in this work.

## 3. Conclusions

In the present work we have calculated the electrostatic interaction between two point charges and between two dipoles placed inside a lipid membrane. Generalizing previous works for a single interface [31,32,33], we have modeled the membrane as a dielectric slab of finite thickness immersed in an aqueous solution containing monovalent anions and cations. Based on the linearized form of the Poisson–Boltzmann approach (known as the Debye-Hückel model), we have formulated integral representations for interacting point charges located at arbitrary positions inside the dielectric slab. The resulting expression, Equation (6) (the main result of this work), bears the interplay between the mismatch in dielectric constants and the screening promoted by the polarization of the electrolyte. We have discussed limiting cases where analytic solutions are available and investigated some of the behaviors between these limiting cases numerically. We have also used our integral representation of the electrostatic potential produced by a single point charge inside the dielectric slab to describe the interaction between arbitrarily oriented dipoles. This too leads to an integral representation of the interaction. More complex cases, such as interacting charge distributions or interacting electric multipoles, can, in principle, also be investigated using the linear formalism employed in this work. Another extension is the addition of mobile surface charges (located at the two interfaces), which represent charged lipid headgroups. If mobile, they will give rise to yet another polarization mechanism [44] that complements the two investigated in the present work.

We reiterate the key assumption that allows us to derive Equation (6) is the use of linear electrostatics, which becomes valid in the limit of small electrostatic potentials everywhere inside the aqueous solution. This neglects all non-linear effects, including the ion size, dielectric saturation and (as always for mean-field models) ion-ion correlations. However, the location of the point charges in our model inside the dielectric slab renders the use of linear electrostatics a much better approximation than their placement into the salt-containing aqueous solution. It is, nevertheless, in principle possible to consider the inclusion of previously developed models for electrolytes of higher ion concentrations [45,46,47] or dielectric media that allow for field-dependent saturation effects [48,49,50,51] into the current formalism.

## Figures and Tables

**Figure A1:**
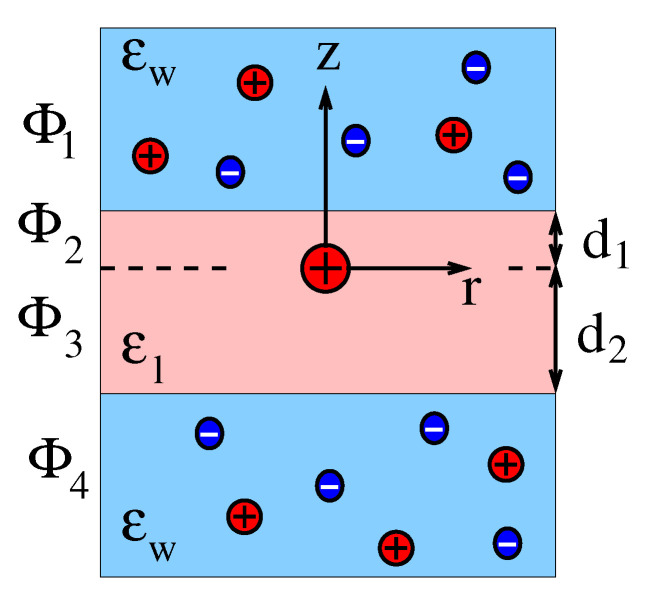
The origin of a cylindrical coordinate system {r,ϕ,z} is placed at the location of a point charge *q*, with distances $d_{1}$ and $d_{2}$ away from the two interfaces of the dielectric slab, which has a thickness $d=d_{1}+d_{2}$. The dielectric constant inside the dielectric slab is $\epsilon_{l}$, that inside the sandwiching media is $\epsilon_{w}$. We denote the electric potential in the region $z>d_{1}$ by $\Phi_{1}$, that in the region $d_{1}>z>0$ by $\Phi_{2}$, that in the region $0>z>-d_{2}$ by $\Phi_{3}$, and that in the region $-d_{2}>z>$ by $\Phi_{4}$. Salt is present in the two sandwiching media with bulk concentration $n_{0}$.

**Figure A2:**
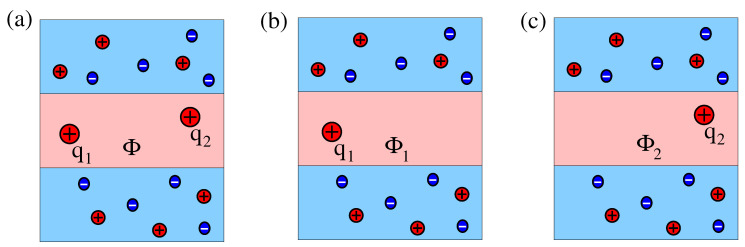
Diagram (**a**) Two point charges, $q_{1}$ and $q_{2}$ located inside a lipid membrane produce an electrostatic potential Φ. Diagram (**b**) We remove the charge $q_{2}$ and denote the potential due to the presence of the remaining charge $q_{1}$ by $\Phi_{1}$. Diagram (**c**) We remove the charge $q_{1}$ and denote the potential due to the presence of the remaining charge $q_{2}$ by $\Phi_{2}$.

## Appendix A

This appendix details the calculation of the electrostatic potential Φ for a the presence of one single point charge *q* located inside the dielectric slab. As the system is cylindrically symmetric, it is convenient to place a cylindrical coordinate system {r,ϕ,z} at the location of the point charge *q* as shown in Figure A1.

Note that the electrostatic potential Φ=Φ(r,z) is independent of the azimuthal angle ϕ. We divide the system into four regions along the *z*-axis and—as illustrated in Figure A1—denote the electrostatic potential by $\Phi_{1}=\Phi_{1}\left(r,z\right)$ for $z>d_{1}$, by $\Phi_{2}=\Phi_{2}\left(r,z\right)$ for $d_{1}>z>0$, by $\Phi_{3}=\Phi_{3}\left(r,z\right)$ for $0>z>-d_{2}$, and by $\Phi_{4}=\Phi_{4}\left(r,z\right)$ for $-d_{2}>z$. These potentials satisfy the differential equations $l_{D}^{2}\nabla^{2}\Phi_{1}=\Phi_{1}$, $\nabla^{2}\Phi_{2}=0$, $\nabla^{2}\Phi_{3}=0$, and $l_{D}^{2}\nabla^{2}\Phi_{4}=\Phi_{4}$ and the boundary conditions

(A1) $$\begin{array}{ccc} \epsilon_{w}{\left(\frac{\partial \Phi_{1}}{\partial z}\right)}_{z=d_{1}}-\epsilon_{l}{\left(\frac{\partial \Phi_{2}}{\partial z}\right)}_{z=d_{1}} & = & 0,\;\epsilon_{l}{\left(\frac{\partial \Phi_{2}}{\partial z}\right)}_{z=0}-\epsilon_{l}{\left(\frac{\partial \Phi_{3}}{\partial z}\right)}_{z=0}=-\frac{q}{\epsilon_{0}}\delta \left(r\right),\\ \epsilon_{l}{\left(\frac{\partial \Phi_{3}}{\partial z}\right)}_{z=-d_{2}}-\epsilon_{w}{\left(\frac{\partial \Phi_{4}}{\partial z}\right)}_{z=-d_{2}} & = & 0. \end{array}$$

where δ(r) denotes the Dirac delta function [52]. At z→±∞ the corresponding potential vanishes. Continuity of the electrostatic potential demands $\Phi_{1}\left(r,z=d_{1}\right)=\Phi_{2}\left(r,z=d_{1}\right)$, $\Phi_{2}\left(r,z=0\right)=\Phi_{3}\left(r,z=0\right)$, and $\Phi_{3}\left(r,z=-d_{2}\right)=\Phi_{4}\left(r,z=-d_{2}\right)$. We express the solution of the differential equations in the four regions as

(A2) $$\begin{array}{ccc} \Phi_{1}\left(r,z\right) & = & \frac{1}{2\pi }\int_{0}^{\infty }dk\;k\;J_{0}\left(kr\right)A_{1}e^{-\sqrt{k^{2}+l_{D}^{-2}}z},\;\Phi_{2}\left(r,z\right)=\frac{1}{2\pi }\int_{0}^{\infty }dk\;k\;J_{0}\left(kr\right)\left(A_{2}e^{kz}+A_{3}e^{-kz}\right),\\ \Phi_{3}\left(r,z\right) & = & \frac{1}{2\pi }\int_{0}^{\infty }dk\;k\;J_{0}\left(kr\right)\left(A_{4}e^{kz}+A_{5}e^{-kz}\right),\;\Phi_{4}\left(r,z\right)=\frac{1}{2\pi }\int_{0}^{\infty }dk\;k\;J_{0}\left(kr\right)A_{6}e^{\sqrt{k^{2}+l_{D}^{-2}}z}. \end{array}$$

The three boundary and three continuity conditions specified above fully determine the six constants $A_{i}=A_{i}\left(k\right)$ with i=1…6.

## Appendix B

We demonstrate how the interaction energy *U* between two charges $q_{1}$ and $q_{2}$ is determined from the potential Φ calculated for one single charge, either $q_{1}$ or $q_{2}$. Assume the two charges reside at positions $r_{1}$ and $r_{2}$ somewhere in the system; Figure A2a displays them inside the dielectric slab.

The mean-field free energy *F* of the system according to linearized Poisson–Boltzmann theory is given by

(A3) $$F=\frac{\epsilon_{0}\epsilon_{l}}{2}\int_{l}dv{\left(\nabla \Phi \right)}^{2}+\frac{\epsilon_{0}\epsilon_{w}}{2}\int_{w}dv{\left(\nabla \Phi \right)}^{2}+k_{B}T\int_{w}dv\left[\frac{{\left(n_{+}-n_{0}\right)}^{2}}{2n_{0}}+\frac{{\left(n_{-}-n_{0}\right)}^{2}}{2n_{0}}\right].$$

Here, the first term is the energy of the electrostatic field inside the dielectric slab (region “*l*”, where the dielectric constant is $\epsilon_{l}$), the second term is the energy of the electrostatic field inside the aqueous solution (region “*w*”, where the dielectric constant is $\epsilon_{w}$), and the third term is the demixing entropy of the salt ions on the level of the linearized Poisson–Boltzmann theory, expressed in terms of the local cation and anion concentrations, $n_{+}$ and $n_{-}$, respectively. Moreover, recall that $n_{0}$ is the bulk salt concentration. Subject to the electrostatic potential Φ fulfilling the Laplace equation $\nabla^{2}\Phi =0$ inside the dielectric slab and the linearized Poisson–Boltzmann equation $l_{D}^{2}\nabla^{2}\Phi =\Phi$ inside the aqueous solution, minimization of *F* with respect to $n_{+}$ and $n_{-}$ yields the equilibrium distributions $n_{+}=n_{0}\left(1-e\Phi /k_{B}T\right)$ and $n_{-}=n_{0}\left(1+e\Phi /k_{B}T\right)$, which, when inserted back into Equation (A3), gives rise to

(A4) $$F=\frac{\epsilon_{0}\epsilon_{l}}{2}\int_{l}dv{\left(\nabla \Phi \right)}^{2}+\frac{\epsilon_{0}\epsilon_{w}}{2}\int_{w}dv\left[{\left(\nabla \Phi \right)}^{2}+\frac{1}{l_{D}^{2}}\Phi^{2}\right]=\frac{q_{1}}{2}\Phi \left(r_{1}\right)+\frac{q_{2}}{2}\Phi \left(r_{2}\right).$$

Next, assume we remove $q_{2}$ (Figure A2b) and denote the remaining potential due to the presence of $q_{1}$ by $\Phi_{1}\left(r\right)$. Similarly, we may remove $q_{1}$ (Figure A2c) and denote the remaining potential due to the presence of $q_{2}$ by $\Phi_{2}\left(r\right)$. Linearity of our model renders the potential of the composite system (where both $q_{1}$ and $q_{2}$ are present) the sum of the potentials for the individual charges, $\Phi \left(r\right)=\Phi_{1}\left(r\right)+\Phi_{2}\left(r\right)$. Inserting this into Equation (A4) yields the self energies $q_{1}\Phi_{1}\left(r_{1}\right)/2+q_{2}\Phi_{2}\left(r_{2}\right)/2$ and the interaction energy $q_{1}\Phi_{2}\left(r_{1}\right)/2+q_{2}\Phi_{1}\left(r_{2}\right)/2$. According to Green’s reciprocity relation [38], both terms in the interaction energy are the same, implying we can calculate the interaction energy either by $q_{1}\Phi_{2}\left(r_{1}\right)$ or $q_{2}\Phi_{1}\left(r_{2}\right)$. This is indeed how we calculate the interaction energy in Equation (4) from the potential Φ in Appendix A.

## Appendix C

We describe our method to calculate the dipole–dipole interaction energy in Equation (11). However, instead of presenting the formalism for the general case (which leads to cumbersome expressions), we focus on the limit d→∞, which corresponds to a single salt-free medium of dielectric constant $\epsilon_{l}$. The interaction energy is then very simple, $U=\mu_{1}\mu_{2}/\left(4\pi \epsilon_{0}\epsilon_{l}r^{3}\right)$, and our method of derivation is transparent and translates analogously to the case where a dielectric slab is present. We start from the electrostatic potential produced by a single charge $q_{1}$, located at the origin of our cylindrical coordinate system

(A5) $$\Phi \left(r,z\right)=\frac{q_{1}}{4\pi \epsilon_{0}\epsilon_{l}}\int_{0}^{\infty }dk\;J_{0}\left(kr\right)e^{-|k|z}=\frac{q_{1}}{4\pi \epsilon_{0}\epsilon_{l}}\frac{1}{\sqrt{r^{2}+z^{2}}}.$$

The interaction between two dipoles that are aligned along the *z*-direction and separated by a distance *r* is

(A6) $$U=2q_{2}\left[\Phi (r,0)-\Phi (r,l)\right]=\frac{q_{1}q_{2}}{2\pi \epsilon_{0}\epsilon_{l}}\int_{0}^{\infty }dk\;J_{0}\left(kr\right)\left(1-e^{-kl}\right),$$

where l>0 must be sufficiently small. The expansion of the integrand in Equation (A6) with respect to small *l* must be carried out carefully because *k* runs from zero to infinity. Hence, we replace *l* by l+δ (with δ>0) and, prior to taking the limit δ→0, we perform a series expansion with respect to *l* up to second order and carry out the integral. This yields

(A7) $$U=\frac{q_{1}q_{2}}{2\pi \epsilon_{0}\epsilon_{l}}\lim_{\delta \to 0}\int_{0}^{\infty }dk\;J_{0}\left(kr\right)\left[1-e^{-k\delta }\left(1-kl+k^{2}l^{2}\right)\right].$$

The integrals of zeroth and first order in *l* vanish, and for the second order we obtain Equation (12). The same method can be applied when the dielectric slab is present, which leads to Equation (11).

## Appendix D

As already in Appendix C, we find it most instructive to present the interaction between the dipoles, which now can have arbitrary orientation, for the case of a uniform, salt-free medium of dielectric constant $\epsilon_{l}$. This corresponds to the limit d→∞ in Equation (14). We consider the four vectors

(A8) $${\vec{r}}_{ij}=\left(\begin{array}{c} x_{ij}\\ y_{ij}\\ z_{ij} \end{array}\right)=\left(\begin{array}{c} 0\\ 0\\ r \end{array}\right)+\frac{l}{2}\left[{\left(-1\right)}^{i+1}\left(\begin{array}{c} \sin\theta_{1}\cos\phi_{1}\\ \sin\theta_{1}\sin\phi_{1}\\ \cos\theta_{1} \end{array}\right)+{\left(-1\right)}^{j}\left(\begin{array}{c} \sin\theta_{2}\cos\phi_{2}\\ \sin\theta_{2}\sin\phi_{2}\\ \cos\theta_{2} \end{array}\right)\right]$$

that connect each end of one dipole to each end of the other, with i=1,2 and j=1,2. The interaction energy between the two dipoles can then be written as

(A9) $$U=\frac{q_{1}q_{2}}{4\pi \epsilon_{0}\epsilon_{l}}\sum_{i=1}^{2}\sum_{j=1}^{2}\frac{{\left(-1\right)}^{i+j}}{\sqrt{z_{ij}^{2}+x_{ij}^{2}+y_{ij}^{2}}}=\frac{q_{1}q_{2}}{4\pi \epsilon_{0}\epsilon_{l}}\int_{0}^{\infty }dk\sum_{i=1}^{2}\sum_{j=1}^{2}{\left(-1\right)}^{i+j}J_{0}\left(kz_{ij}\right)e^{-k\sqrt{x_{ij}^{2}+y_{ij}^{2}}}.$$

Expanding the integrand with respect to *l* leads (up to quadratic order) to

(A10) $$U=\frac{q_{1}q_{2}l^{2}}{4\pi \epsilon_{0}\epsilon_{l}}\lim_{\delta \to 0}\int_{0}^{\infty }dk\;k\;e^{-k\delta }\left\{kJ_{0}\left(kr\right)\left[\cos\theta_{1}\cos\theta_{2}-\cos\left(\phi_{1}-\phi_{2}\right)\sin\theta_{1}\sin\theta_{2}\right]-\frac{J_{1}\left(kr\right)}{r}\cos\theta_{1}\cos\theta_{2}\right\}.$$

Noting the two integrals

(A11) $$\lim_{\delta \to 0}\int_{0}^{\infty }dk\;k\;e^{-k\delta }J_{1}\left(k\right)=1,\;\lim_{\delta \to 0}\int_{0}^{\infty }dk\;k^{2}\;e^{-k\delta }J_{0}\left(k\right)=-1,$$

and inserting the dipole moments $\mu_{1}=q_{1}l$ as well as $\mu_{2}=q_{2}l$, the interaction energy *U* in Equation (A10) becomes identical to Equation (15).
